# Effects of *Caulerpa lentillifera* on Growth Performance, Antioxidant Capacity and Intestinal Microbiota of *Litopenaeus vannamei*

**DOI:** 10.3390/biology14121738

**Published:** 2025-12-04

**Authors:** Hong Liang, Jialin Tian, Yun Wang, Yafei Duan, Jun Wang, Chuanpeng Zhou, Zhong Huang

**Affiliations:** 1 College of Fisheries and Life Sciences, Shanghai Ocean University, Shanghai 201306, China; lianghong99001122@163.com; 2 Key Laboratory of Aquatic Product Processing, Ministry of Agriculture and Rural Affairs, South China Sea Fisheries Research Institute, Chinese Academy of Fishery Sciences, Guangzhou 510300, China; jltian2023@163.com (J.T.); duanyafei@scsfri.ac.cn (Y.D.); jwang@scsfri.ac.cn (J.W.); zhoucp@scsfri.ac.cn (C.Z.); 3 Key Laboratory of Efficient Utilization and Processing of Marine Fishery Resources of Hainan Province, Hainan Engineering Research Center of Deep-Sea Aquaculture and Processing, Sanya Tropical Fisheries Research Institute, Sanya 572000, China; 4 Guangdong Provincial Key Laboratory of Fishery Ecology and Environment, South China Sea Fisheries Research Institute, Chinese Academy of Fisheries Sciences, Guangzhou 510300, China; 5 Shenzhen Base of South China Sea Fisheries Research Institute, Chinese Academy of Fishery Sciences, Shenzhen 518121, China; huangzhongnhs@163.com

**Keywords:** *Litopenaeus vannamei*, *Caulerpa lentillifera*, growth performance, antioxidant capacity, intestinal microbiota

## Abstract

To investigate the effects of supplementing the diet with *Caulerpa lentillifera* on the growth performance, antioxidant capacity, and intestinal microbiota of *Litopenaeus vannamei*, a total number of 600 healthy shrimp, each having an average body weight of 2.45 ± 0.12 g, were allocated randomly into five experimental groups. These groups comprised a control group receiving a basal diet (Ctrl) and four treatment groups receiving the basal diet supplemented with varying concentrations of dried *C. lentillifera* powder: 2.5% (CL1), 5% (CL2), 7.5% (CL3), and 10% (CL4). Each group consisted of four replicates, with 30 shrimp per replicate. The experimental duration was 56 days. The findings demonstrated that supplementing the diet with 5–7.5% *C. lentillifera* enhanced growth performance and significantly increased the crude protein content in whole shrimp (*p* < 0.05). Based on the feed conversion ratio (FCR) and protein efficiency ratio (PER) metrics, the optimal inculusion range of *C. lentillifera* in *L. vannamei* feed was determined to be 5.25–7.15%. Simultaneously, there was a significant increase in the activities of total superoxide dismutase (T-SOD), glutathione peroxidase (GPx), and peroxidase (POD) enzymes in the hepatopancreas, accompanied by a substantial reduction in malondialdehyde (MDA) levels (*p* < 0.05). Dietary supplementation with 5–7.5% *C. lentillifera* induced a numerical elevation of the relative expression levels of antioxidant and protein synthesis genes, although no statistically significant differences were detected among all groups (*p* > 0.05). Analyses of intestinal microbiota sequencing revealed that the addition of 5–7.5% *C. lentillifera* improved the intestinal microbiota’s composition and structural characteristics with a focus on metabolic pathways. In conclusion, the inclusion of dietary *C. lentillifera* positively influenced growth performance, antioxidant capacity, and the intestinal microbiota of *L. vannamei*. However, excessively high-dose dietary levels may have adverse effects on shrimp; thus, a dietary inclusion level of 5.25–7.15% *C. lentillifera* is recommended.

## Simple Summary

The continuous expansion of aquaculture operations has exacerbated the prevalence and severity of shrimp diseases, resulting in significant economic losses for the shrimp farming industry. Although antibiotics can offer certain advantages in shrimp production, their negative impacts necessitate careful evaluation. Consequently, the development of innovative, eco-friendly feed additives has emerged as a crucial area of focus in aquatic nutrition research. While research on dietary *Caulerpa lentillifera* in mammals suggests its potential as an effective functional aquatic feed additive due to its antioxidant, immunomodulatory, anti-inflammatory, and intestinal microbiota-regulating properties, its effects on shrimp remain largely unexplored. This study investigated the impact of dietary *C. lentillifera* on growth performance, antioxidant capacity, and intestinal microbiota in *Litopenaeus vannamei*. The findings demonstrate that the inclusion of *C. lentillifera* in *L. vannamei* diets is both viable and advantageous. *C. lentillifera* was found to enhance growth performance, antioxidant capacity, and intestinal microbiota composition, with an optimal supplementation rage of 5.25 to 7.15%. The outcomes of this research build a theoretical foundation for the efficient application of *C. lentillifera* in *L. vannamei* farming and pave the way for subsequent investigations into its metabolic processes.

## 1. Introduction

Aquaculture constitutes a fundamental component of the global food system, serving as a crucial source of animal protein for billions of individuals worldwide [1]. Boasting fast growth rate, high protein content, and excellent environmental adaptability, *Litopenaeus vannamei* has emerged as a dominant species in the global aquaculture industry [2,3,4]. *L. vannamei* achieved a production volume of approximately 6 million tons in 2022, marking an increase of about 5% from the previous year [5]. However, the intensification of aquaculture practices has heightened shrimp vulnerability to oxidative stress, pathogenic attacks and intestinal microecological imbalance. Concurrently, the excessive application of antibiotics and chemical additives in conventional feeds has precipitated a range of issues, including drug resistance, environmental pollution, and concerns regarding product safety [6]. Consequently, the development of novel environmentally friendly feed additives has emerged as a critical focus within aquatic nutrition research [7].

Macroalgae predominantly thrive in global coastal zones, thus circumventing competition for arable land resources. Historically, macroalgae have served as a versatile resource in Asia, with a long-standing tradition of utilization as food, feed, and medicinal materials dating back to ancient times [8]. Exposed to high light intensities and elevated oxygen concentrations, seaweeds are constantly challenged by free radicals and potent oxidizing agents [9]. Paradoxically, despite these harsh conditions, seaweed cells remain structurally intact, and their oxidative stability during photosynthetic storage persists, which strongly implies the existence of robust antioxidative defense mechanisms and compounds within these cells, safeguarding them against oxidative stress. Macroalgae thalli are abundant in dietary fiber, with polysaccharides serving as prebiotics [10]. *Caulerpa lentillifera*, a species of green macroalgae, is widely distributed in subtropical and tropical regions and can be consumed directly [11]. Naturally, *C. lentillifera* is abundant inabundant in sulfated polysaccharides, phenolic compounds, proteins, amino acids, lipids, fatty acids, minerals, vitamins, and pigments [12]. Lipid extracts from *C. lentillifera* exhibit the ability to scavenge oxidized free radicals and demonstrate significant antioxidant capacity in vitro [13]. Polyphenol extracts show strong hydrogen peroxide scavenging activity [14]. Studies on mammals have demonstrated that *C. lentillifera* or its extracts possess antioxidant, immunomodulatory, anti-inflammatory, hypoglycemic, hypolipidemic, and intestinal microbiota-regulating properties [12,15,16,17,18,19]. In aquatic animals, polysaccharides from *C. lentillifera* not only improve the growth performance and body protein content of *L. vannamei*, and enhance their immune response optimize the intestinal microbiota composition by increasing the abundance of beneficial bacteria and reducing harmful bacteria [20], but also mitigate oxidative stress-induced detrimental effects in *Danio rerio* [21]. Given these attributes, *C. lentillifera* holds potential as a highly effective functional aquafeed additive, particularly in enhancing antioxidant activity and promoting intestinal health.

However, the role of non-extractable components in contributing to antioxidant capacity and intestinal microbiota remains unclear due to limitations in the extraction process. Drying and grinding algae into powder appears to be a more favorable processing method for preserving the integrity of bioactive components and enhancing nutrient synergy. *C. lentillifera* powder has been shown to effectively enhance the growth performance of *Penaeus monodon* and *Chanos chanos* [22,23]. Conversely, the scientific literature regarding the application of *C. lentillifera* powder in *L. vannamei* remains notably scarce. The extant body of research on this topic is limited to a solitary investigation that employed a singular supplementation level of 50 g kg^−1^ [8]. While this study provided evidence that *C. lentillifera* powder could augment the immunological defenses and antioxidant capabilities of *L. vannamei*, it notably lacked an elucidation of the dose–response relationship. Additionally, the study did not comprehensively explore the profound impacts of *C. lentillifera* powder on the intestinal microbiota of *L. vannamei*, leaving significant knowledge gaps in these critical areas.

Consequently, this research intends to assess the impact of *C. lentillifera* on the growth, whole body composition, antioxidant capacity and intestinal microbiota of *L. vannamei*. The results of this study help clarify the regulatory effects of different dietary *C. lentillifera* powder supplementation levels on the antioxidant capacity and intestinal microbiota of *L. vannamei*, define its optimal supplementation range, and deeply elaborate on the correlation mechanism between antioxidant mechanisms and intestinal microbiota regulation.

## 2. Materials and Methods

### 2.1. Experimental Diets

The raw materials were initially crushed and then sieved using an 80-mesh sieve. *C. lentillifera* (the nutritional components were measurement by Zhou [24], and detailed in Table S1) underwent a drying process, following by crushing and sieving through an 80-mesh sieve to produce a dry algae powder. Subsequently, five experimental diets were formulated by incorporating varying concentrations of *C. lentillifera* powder into the basal diet of *L. vannamei* at levels of 0% (Ctrl), 2.5% (CL1), 5% (CL2), 7.5% (CL3), and 10% (CL4). The specific feed formulations and their constituent analyses are detailed in Table 1. The ingredients were thoroughly mixed according to the specified formulations, combined with water, and extruded into thin strands having diameters of 1.0 mm and 1.5 mm using a double-screw bar extruder (F-26, South China University of Technology, Guangzhou, China). The resulting feeds were then pelletized using a pelletizer (G-500, South China University of Technology, Guangzhou, China), subjected to heating in an oven at 90 °C for 90 min, air-dried to approximately 10% moisture content, and subsequently stored in a freezer at 4 °C for preservation.

### 2.2. Experimental Animals and Aquaculture Management

All *L. vannamei* specimens were temporarily reared for a duration of one week at the Shenzhen base of the South China Sea Fisheries Research Institute of the Chinese Academy of Fisheries Sciences. The *L. vannamei*, with an initial mean weight of (2.45 ± 0.12) g, were randomly allocated into twenty 500 L cylindrical fiberglass thanks, with each tank containing 30 shrimp. The experiment was structured into five distinct treatment groups, each comprising four replicates. Throughout the culture period, residual material in the bait trays was collected and dried one hour post-feeding to quantify the feed intake. Additionally, Shrimp mortality and body weight per container were documented systematically. The aquaculture system utilized filtered seawater, which was siphoned from the bottom daily, with one-third of the water volume being replaced weekly. Throughout the experimental phase, water temperature was kept at 27.8 ± 3.9 °C, salinity approximated 29.5 ± 0.7, pH stabilized at 8.0 ± 0.1, ammonia nitrogen concentration remained below 0.05 mg L^−1^, and dissolved oxygen levels exceeded 6.0 mg L^−1^.

### 2.3. Sample Collection and Processing

Prior to sampling, *L. vannamei* were subjected to a fasting period for 24 h. During sampling, the total number of shrimp and their cumulative weight in each container were chronicled. Shrimp were picked randomly from each tank, and their body surfaces were disinfected with 75% alcohol. Hepatopancreas tissues from five randomly selected shrimp per container were excised and preserved in cryotubes with RNA preservation solution for subsequent analyses of enzyme activity and quantitative real-time polymerase chain reaction (qPCR) assays. Five shrimp intestines were placed in 2 mL cryogenic tubes and promptly submerged in liquid nitrogen for subsequent analysis of intestinal microbiota. Subsequently, all samples were moved to a −80 °C freezer for storage. Additionally, five whole shrimp were randomly selected from each container to assess routine nutrient content and were stored in a −20 °C freezer.

### 2.4. Growth Performance and Feed Utilization

The survival rate (SR, %), weight gain rate (WGR, %), specific growth rate (SGR, % day^−1^), feed conversion ratio (FCR), and protein efficiency ratio (PER) were evaluated. The growth parameters were calculated using the formulas listed below:

SR (%) = 100 × final survival number of shrimp/initial number of shrimp(1)

WGR (%) = 100 × (W*_t_* − W*_i_*)/W*_i_* (2)

SGR (% day^−1^) = 100 × (Ln W*_t_* − Ln W*_i_*)/*t* (3)

FCR = W*_f_*/(W*_t_* − W*_i_*) (4)

PER = body weight gain/protein intake(5)

where W*_t_*, represents the average final body weight (g) at feeding time *t* (days), W*_i_* is the average initial body weight (g), W*_f_* is the total feed consumption as dry matter (g).

### 2.5. Nutritional Composition of the Whole Shrimp

The nutrient composition of the feeds and whole shrimp was assayed following the methods specified by the Association of Official Analytical Chemists (AOAC, 1997) [25]. Moisture content was assayed by atmospheric pressure drying method. Samples were placed in a preheated 105 °C constant-temperature oven until constant weight. Ash content was measured via the high-temperature ashing using a muffle furnace (Model FO610C, Yamato Scientific Co., Ltd., Tokyo, Japan). Samples were first charred over a low flame on an electric hot plate, then transferred into the muffle furnace. The furnace was set at 550 °C for insulated ashing until the organic matter in the samples was completely carbonized. Crude protein content was assessed in accordance with the Kjeldahl nitrogen determination principle using a fully automatic Kjeldahl nitrogen analyzer (Model Gerhardt VAPODEST500, C. Gerhardt GmbH & Co.KG, Hamburg, Germany). The analyzer automatically performed the entire process of sample digestion, distillation, and titration. Crude lipid content was determined by the Soxhlet extraction method with a Soxhlet extraction system (Model Gerhardt Soxtherm, C. Gerhardt GmbH & Co.KG, Hamburg, Germany). Anhydrous diethyl ether was used as the extraction solvent, and the lipid components in the samples were extracted via solvent reflux.

### 2.6. Enzyme Activity Assay

The hepatopancreas was homogenized by mixing it with phosphate-buffered saline (PBS) at a 1:9 ratio. The mixture was then centrifuged at 3000 rpm min^−1^ for 10 min at 4 °C, and the resulting supernatant was collected afterward. The activities of total protein (TP), total antioxidant capacity (T-AOC), total superoxide dismutase (T-SOD), catalase (CAT), glutathione peroxidase (GPx), peroxidase (POD) activities, as well as malondialdehyde (MDA) levels, were determined with kits supplied by Nanjing Jiancheng Bioengineering Institute (Nanjing, China).

### 2.7. RNA Extraction and qPCR Assay

Hepatopancreas-derived total RNA was isolated using Trizol reagent (Invitrogen, Shanghai, China). The Evo M-MLV Reverse Transcription Premix Kit (Accurate Biotechnology Code No. AG11728, Changsha, China) was employed to synthesize first-strand cDNA, following the manufacturer’s guidelines. Primer sequences utilized in this study are detailed in Table S2 and were synthesized by Sangon Biotech (Shanghai, China). The qPCR technique was conducted using the SYBR Green method, with the SYBR Green Pro Taq HS qPCR premix kit (Accurate Biotechnology Code No. AG11701, Changsha, China). PCR amplified using a PCR detection system (Heal Force CG-02, Shanghai, China). The ∆∆Ct for each sample in the experimental group was calculated by subtracting the mean ∆Ct of the control group from the ∆Ct of that individual experimental sample. Fold changes were derived using the formula 2^−∆∆Ct^. Quantitative results were analyzed by means of the equation provided below:

∆Ct = Target gene Ct value − Reference gene Ct value

∆∆Ct value = ∆Ct value of treatment group − ∆Ct mean value of all control groups

### 2.8. Intestinal Microbial Community Analysis

Intestinal microbiota samples were used for genomic DNA extraction with the OMEGA Soil DNA Kit (M5635-02, Omega Bio-Tek, Norcross, GA, USA), and the extracted DNA was stored at −20 °C. Primers 338F (5′-ACTCCTACGGGGAGGCAGCA-3′) and 806R (5′-GGACTACHVGGGGTWTCTAAT-3′) were employed to amplify the V3-V4 region of the bacterial 16S rRNA gene. After purification and recovery of the PCR products with magnetic beads, fluorescence quantification was performed via the Quant-iT PicoGreen dsDNA Assay Kit (Eugene, OR, USA) on a Bio Tek FLx800 Microplate reader (Winooski, VT, USA). Subsequent library preparation for sequencing utilized Illumina’s TruSeq Nano DNA LT Library Prep Kit (San Diego, CA, USA), with paired-end sequencing performed on a NovaSeq 6000 sequencer using the NovaSeq 6000 SP kit (San Diego, CA, USA). Microbiota analyses were completed at Suzhou Panomix Biomedical Tech Co., Ltd. (Suzhou, China).

### 2.9. Statistical Analysis

The experimental data were expressed as mean ± standard deviation (SD). Before conducting statistical analyses, the normality of the data distribution was evaluated using the Shapiro–Wilk test, and the homogeneity of variance was assessed via Levene’s test. Inter-group mean differences were analyzed via one-way analysis of variance (ANOVA), followed by Tukey’s honestly significant difference (HSD) test for post hoc multiple comparisons; statistical significance was defined at *p* < 0.05. Inter-group comparisons were performed using a one-way analysis of variance (ANOVA) to identify significant differences among group means, with Tukey’s honestly significant difference (HSD) test employed for post hoc multiple comparisons. If parametric test assumptions were unmet, the non-parametric Kruskal–Wallis rank-sum test was used instead, with subsequent non-parametric pairwise comparisons performed via Dunn’s test (Bonferroni-corrected) to control the family-wise error rate. All statistical analyses were conducted using Statistical Package for the Social Sciences (SPSS) software (Version 27.0; IBM Corp., Chicago, IL, USA). Correlations between variables were analyzed on the BioDeep Platform (https://www.biodeep.cn accessed on 20 June 2024) using the Pearson correlation method.

## 3. Results

### 3.1. The Growth Performance and Nutrient Composition

The growth performance of shrimp was assessed, as detailed in Table 2. Initial body weight exhibited no significant differences across all groups (*p* > 0.05), suggesting an appropriate selection of experimental shrimp. The CL1 group demonstrated the highest SR, while the CL3 group demonstrated the highest WGR and SGR, along with the lowest FCR. The CL2 group recorded the highest PER. The FCR initially decreased and subsequently increased with the incremental addition of *C. lentillifera*, whereas the PER initially increased and subsequently decreased. These results suggest that *C. lentillifera* enhances the shrimp’s efficient utilization of feed, facilitating the complete conversion of dietary protein into animal tissue.

**Figure 1:**
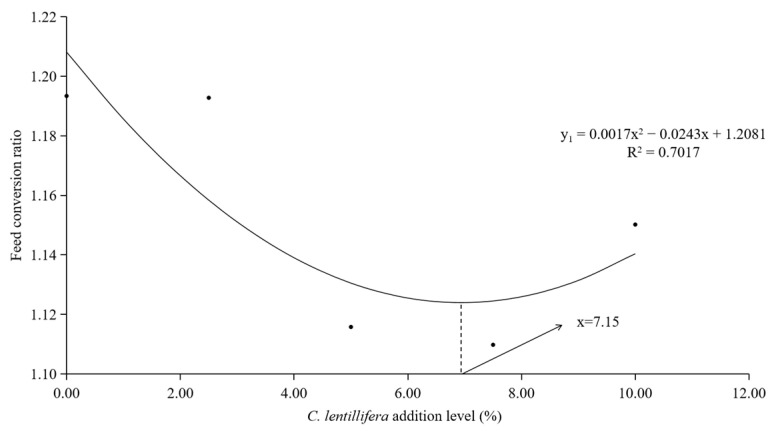
The correlation between the FCR and the inclusion levels of *C. lentillifera* in the diets of *L. vannamei*.

**Figure 2:**
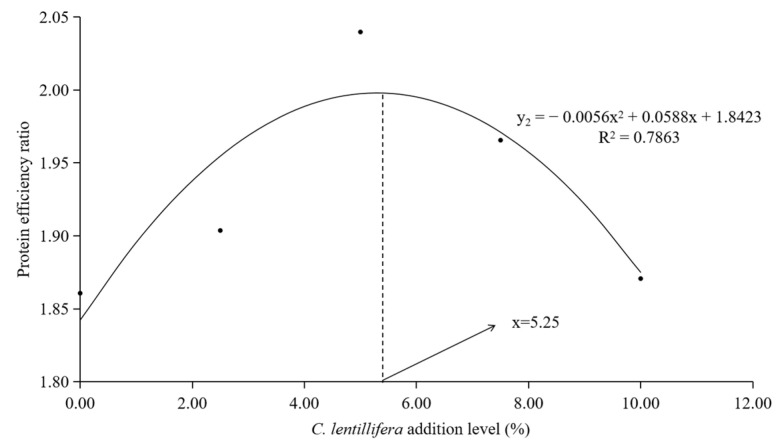
The correlation between the PER and the inclusion levels of *C. lentillifera* in the diets of *L. vannamei*.

The quadratic regression model was employed to assess the optimal inclusion level of *C. lentillifera* in the diet of *L. vannamei*. The quadratic regression equation describing the relationship between the FCR of *L. vannamei* and the inclusion level of *C. lentillifera* in the diet is given by y_1_ = 0.0017x^2^ − 0.0243x + 1.2081 (R^2^ = 0.7017) (Figure 1). Similarly, the quadratic regression equation for the protein efficiency ratio is y_2_ = −0.0056x^2^ + 0.0588x + 1.8423 (R^2^ = 0.7863) (Figure 2). These equations suggest that the optimal inclusion level of *C. lentillifera* in the diet of *L. vannamei* ranges from 5.25 to 7.15%.

The impact of *C. lentillifera* on the nutritional composition of whole shrimp was evaluated (Table 3). The results indicated no significant differences in crude lipid, moisture, and ash content across all groups (*p* > 0.05). However, crude protein levels were significantly elevated in the CL1 to CL3 groups in comparison with the Ctrl group (*p* < 0.05), with the peak level observed in the CL1 group.

### 3.2. Activity of Antioxidant Enzymes in Hepatopancreas

The activities of antioxidant enzymes in the hepatopancreas of *L. vannamei* are detailed in Table 4. In summary, the activities of antioxidant enzymes exhibited an initial increase followed by a decrease with escalating doses of *C. lentillifera*. Notable, the CL3 group exhibited the highest T-AOC and CAT activities, although no significant differences were observed between groups (*p* > 0.05). T-SOD activity in the CL1–CL4 groups was significantly elevated in comparison with the Ctrl group (*p* < 0.05), while GPx activity in the CL1–CL3 groups was significantly higher compared to both the Ctrl and CL4 groups (*p* < 0.05). POD activity showed an upward trend with increased levels of algal powder supplementation, with significantly higher levels observed in the CL3 and CL4 groups compared to in the Ctrl group (*p* < 0.05). The MDA content initially decreased and then increased, with the CL3 group exhibiting significantly lower MDA than in the Ctrl, CL1, and CL4 groups.

### 3.3. Relative Expression Levels of Antioxidant Genes in Hepatopancreas

**Figure 3:**
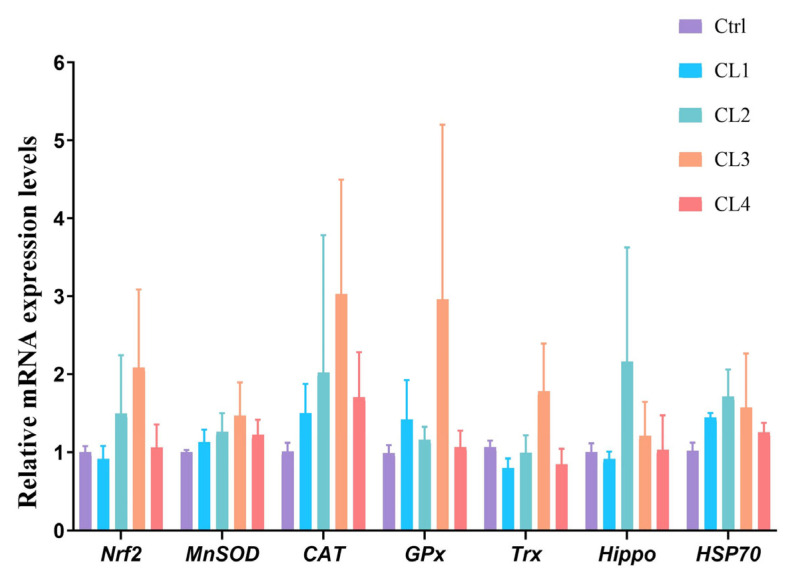
Effects of *C. lentillifera* supplementation on relative expression of antioxidant genes in the hepatopancreas of *L. vannamei.* Bars denote mean ± SD (*n* = 4).

Figure 3 depicts the relative expression levels of antioxidant genes in the hepatopancreas of *L. vannamei*. Upon the supplementation of *C. lentillifera* powder, the relative expression levels of these antioxidant genes in the hepatopancreas initially rose and subsequently declined. In comparison to the Ctrl group, the relative expression levels of nuclear factor erythroid 2-related factor 2 (*Nrf2*), *MnSOD*, *CAT*, *GPx*, thioredoxin (*Trx)*, *Hippo*, and heat shock protein 70 (*HSP70)* were numerically increased in the CL2 and CL3 groups; however, no statistically significant differences were detected in these parameters across all groups.

### 3.4. Relative Expression Levels of Protein Synthesis Genes in Hepatopancreas

**Figure 4:**
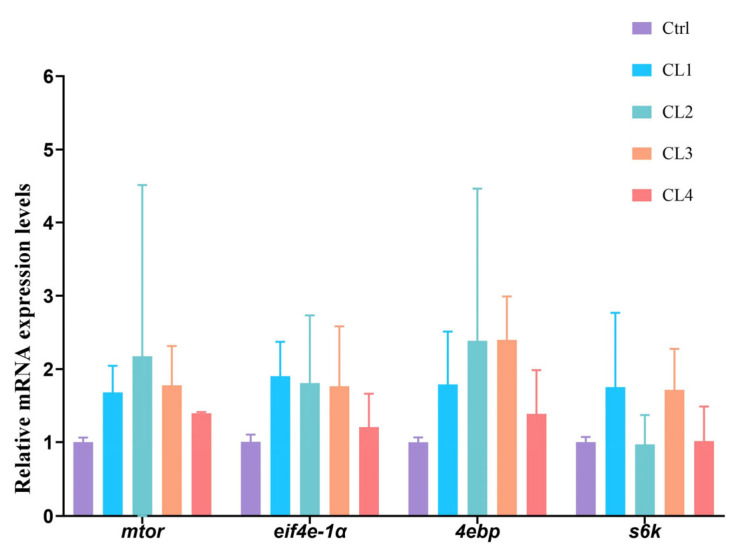
Effects of *C. lentillifera* supplementation on relative expression of protein synthesis genes in the hepatopancreas of *L. vannamei.* Bars denote mean ± SD (*n* = 4).

Figure 4 depicts the relative expression levels of protein synthesis genes in the hepatopancreas of *L. vannamei* following the addition of *C. lentillifera* powder. Initially, the relative expression levels of these protein synthesis genes increased, followed by a subsequent decrease. Compared with the Ctrl group, the relative expression levels of the mammalian target of rapamycinsuper (*mtor)*, eukaryotic initiation factor 4E 1a (*eif4e-1a)*, and 4E-binding protein (*4ebp)* genes were numerically increased in the CL1, CL2, and CL3 groups, whereas the relative expression level of the ribosomal protein S6 kinase (*s6k*) gene was numerically increased in the CL1 and CL3 groups. Nonetheless, no significant differences were observed in these parameters across all groups.

### 3.5. Pearson Correlation-Based Data Analysis

**Figure 5:**
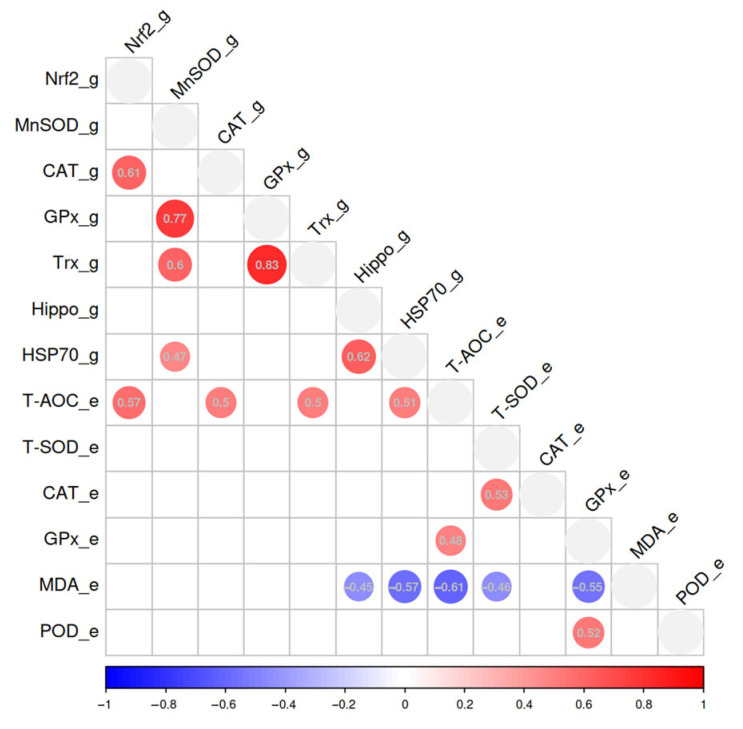
Karl–Pearson correlation plots between antioxidant-related enzyme (_e) activities and gene (_g) indices.

Pearson correlation analysis was conducted to investigate the relationship between antioxidant-related enzyme activities and gene indices, as depicted in Figure 5. The correlation analysis of gene expression revealed a positive correlation between *CAT* and *Nrf2* (r = 0.61). Additionally, *MnSOD* demonstrated positive correlations with *GPx*, *Trx*, and *HSP70* (r = 0.47–0.77), while *GPx* was positively correlated with *Trx* (r = 0.83). Furthermore, *Hippo* demonstrated a positive correlation with *HSP70* (r = 0.62). In terms of antioxidant enzyme activities, T-AOC was positively correlated with GPx (r = 0.48), T-SOD with CAT (r = 0.53), and POD with GPx (r = 0.52). Conversely, MDA content showed negative correlations with T-AOC, T-SOD, and GPx (r = −0.61–0.46). The correlation analysis between antioxidant genes and enzyme activity indicated that T-AOC activity was positively correlated with the relative expressions of *Nrf2*, *CAT*, *Trx* and *HSP70* (r = 0.47–0.57), whereas MDA content was negatively correlated with the relative expressions of *Hippo* and *HSP70* (r = −0.57–−0.45).

**Figure 6:**
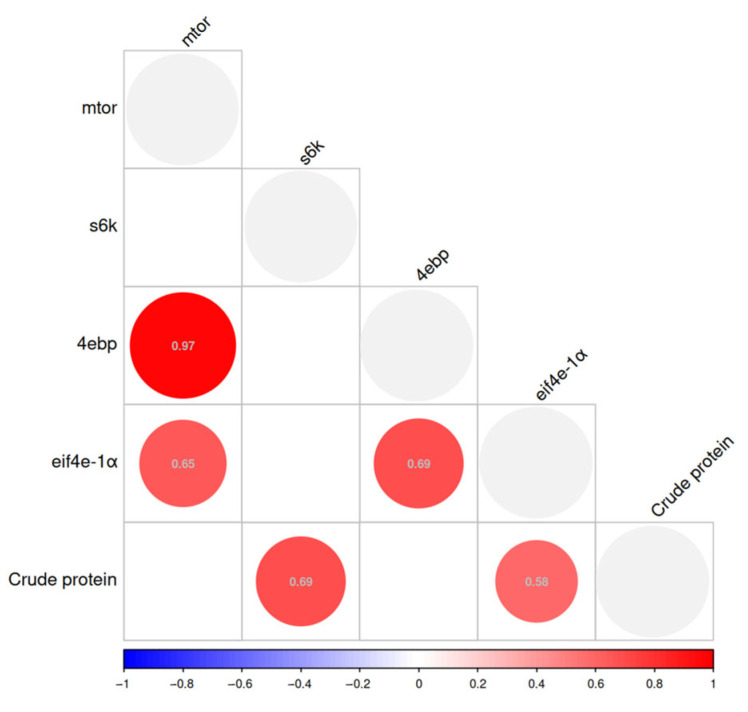
Karl–Pearson correlation plots between protein synthesis gene indices and crude protein content.

Pearson correlation analysis was conducted to investigate the relationship between protein synthesis gene indices and crude protein content, as depicted in Figure 6. The correlation analysis revealed that *mtor* exhibited a positive correlation with *4ebp* and *eif4e-1a* (r = 0.65–0.97). Additionally, *4ebp* was positively correlated with *eif4e-1a* (r = 0.69). Furthermore, the whole shrimp crude protein content demonstrated a positive correlation with *s6k* and *eif4e-1a* (r = 0.58–0.69).

### 3.6. Alterations in Intestinal Microbiota

#### 3.6.1. Microbial Diversity and Composition Changes

**Figure 7:**
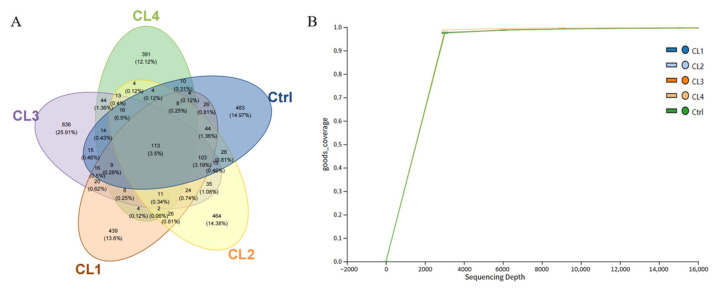
Composition and rationality analysis of ASVs. (**A**): Venn Diagram; (**B**): Good’s nonparametric Coverage estimator.

Amplicon sequence variants (ASVs) of the intestinal microbiota were analyzed, as illustrated in Figure 7A. A total of 812,606 high-quality sequences were obtained from the intestine microbiota of *L. vannamei*, with sequence lengths ranging from 246 to 433 bp, and 99.95% of the sequences falling between 405 and 431 bp. Through 16S rDNA high-throughput sequencing, 3327 ASVs were recognized, of which 113 ASVs were common across all samples. Notably, the CL4 group samples showed the fewest unique ASVs, whereas the CL3 group samples showed the most unique ASVs. As depicted Figure 7B, the rarefaction curves increased rapidly and approached a saturated plateau for all samples, indicating that the sequencing depth was sufficient.

**Figure 8:**
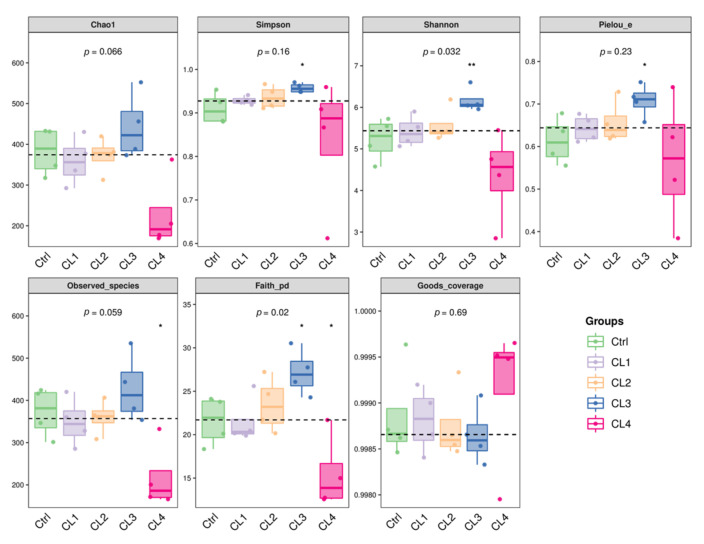
α-diversity indices. Different asterisk symbols represent statistically significant differences: * corresponds to *p* < 0.05, and ** to *p* < 0.01. The dash lines are median lines, representing the median.

In the analysis of Alpha diversity (Figure 8), the indices Simpson and Pielou_e were significantly higher in the CL3 group compared to the remaining groups (*p* < 0.05), Shannon was significantly higher in the CL3 group versus the remaining groups (*p* < 0.01), Observed_species and Faith_pd were significantly lower in the CL3 group versus the remaining groups (*p* < 0.05). No significant differences were observed in Chao1 and Goods_coverage indices among the groups (*p* > 0.05). However, the Chao1 was elevated in the CL3 group, and the Goods_coverage was declined in the CL2 and CL3 groups.

**Figure 9:**
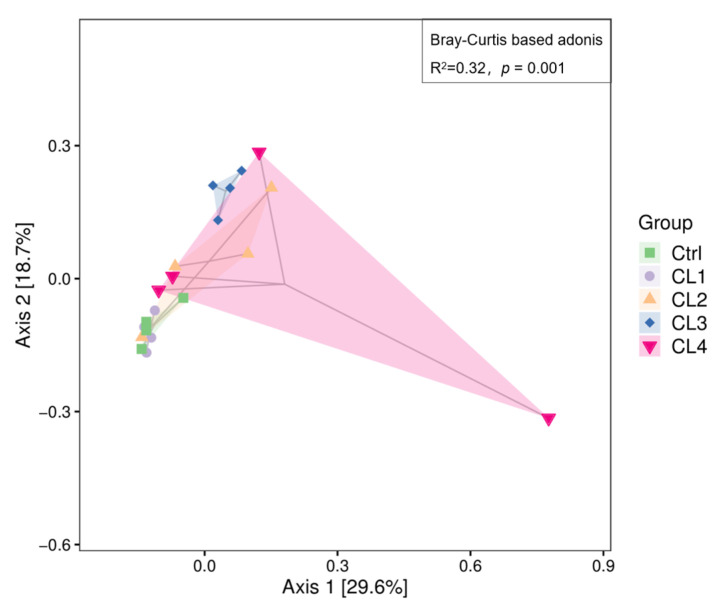
Bray–Curtis based PCoA analysis of samples with 2D ordination plots. The *p*-value in the upper left corner of the figure is derived from the Adonis test, and the R^2^ indicates the degree of explanation of the differences between samples by this grouping method.

Principal Coordinate Analysis (PCoA) of β-diversity based on Bray–Curtis distances revealed differences in microbial community compositions when the CL3 and CL4 groups were compared to the Ctrl group (Figure 9). The Adonis test, based on Bray–Curtis, indicated significant differences in microbial communities between the groups (R^2^ = 0.32, *p* = 0.001).

**Figure 10:**
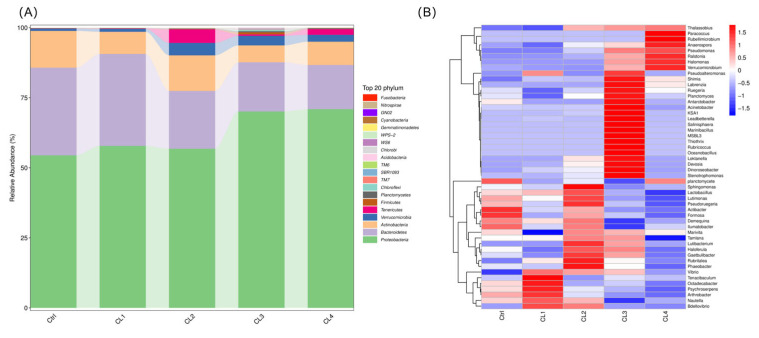
(**A**) Proportional display of the abundance of bacterial phyla. (**B**) Proportional display of the abundance of bacterial genera. Red colors signify increased abundance of bacteria, and blue colors indicate decreased abundance of bacteria.

Although all groups shared of similar dominant bacteria had taxa, their abundance varied. At the phylum level, Proteobacteria, Bacteroidetes, Actinobacteria, Verrucomicrobia constituted the core microbiota (Figure 10A). In comparsion to the Ctrl group, the abundance of Actinobacteria increased with higher levels of *C. lentillifera* supplementation, reaching significantly higher levels in the CL3 and CL4 groups (*p* < 0.05), with the CL4 group exhibiting the highest abundance at 70.94% of the microbiota. Conversely, Bacteroidetes abundance initially increased and subsequently decreased, peaking in the CL1 group at 32.81% of the microbiota, which was significantly higher than in the CL3 and CL4 groups (*p* < 0.05). Notably, Actinobacteria in the CL3 group displayed significantly lower abundance than those in the Ctrl group. (*p* < 0.05). Verrucomicrobia and Tenericutes were primarily detected in the intestinal microbiota of the CL2 group, whereas Firmicutes, Planctomycetes, Chloroflexi, TM7, and SBR1093 were primarily found in the CL3 group. Firmicutes and Chloroflexi were more abundant in the CL3 group compared to the Ctrl group (*p* < 0.05) (Table S3).

At the genus level, *Nautella*, *Ruegeria*, *Demequina,* and *Octadecabacter* were identified as the core microbiota (Figure 10B). In comparison to the Ctrl group, the abundance of *Nautella* initially increased and subsequently decreased, reaching its highest level in the CL1 group, where it constituted 30.88% of the microbiota. Conversely, the abundance of Ruegeria initially decreased and then increased, peaking in the CL3 group at 19.34% of the microbiota. However, no statistically significant differences were observed in the abundance of *Nautella* and *Ruegeria* among the groups (*p* > 0.05). *Demequina* was predominantly present in the Ctrl and CL2 groups, accounting for 8.85% and 9.13% of the microbiota, respectively, which was significantly higher than its abundant in the CL3 group (*p* < 0.05). The abundance of *Octadecabacter* increased and then decreased, with the highest levels observed in the CL1 group, significantly exceeding those in the CL2 and CL4 groups (*p* < 0.05). *Rubritalea* was significantly more abundant in the CL2 group compared to the Ctrl and CL4 groups (*p* < 0.05). Overall, groups CL1 to CL3 exhibited a greater abundance of dominant taxa relative to the Ctrl group (Table S4).

#### 3.6.2. Intestinal Microbiota Phenotypes Changes

**Figure 11:**
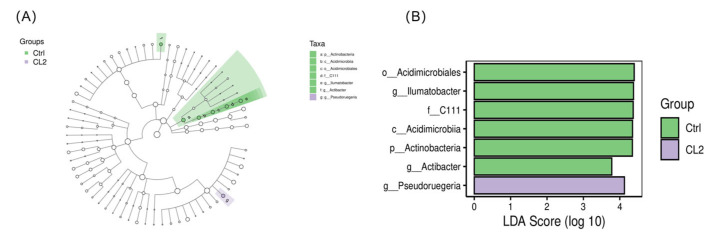
Indicator species analysis of the intestinal microbiota of *L. vannamei* under different feed treatments. (**A**) Lefse cladogram. The cladogram was generated by mapping differences onto a known hierarchical classification tree. Green represents bacteria enriched in the Ctrl group; purple represents bacteria enriched in the CL2 group; white indicates no significant difference. (**B**) Histogram of LDA effect values for marker species. Vertical axes denote categorical units with significant intergroup differences, while horizontal axes display the logarithmic score values from LDA for each categorical unit in bar chart.

Lefse analyses employing effect sizes of linear discriminant analysis (LDA) were employed to examine differences in microbial taxa abundance across groups. The branching diagram illustrated that *Ilumatobacter* was classified from the genus to the phylum level as Actinobacteria. It further demonstrated that *Ilumatobacter* and *Actibacter* could serve as potential biomarkers for the Ctrl group, whereas *Pseudoruegeria* could be employed as a potential biomarker for the CL2 group (Figure 11A). Among bacterial genera with LDA scores exceeding 3.0, both *Ilumatobacter* and *Actibacter* exhibited significant enrichment in the Ctrl group, while *Pseudoruegeria* showed significant enrichment in the CL2 group (Figure 11B).

#### 3.6.3. Prediction of Functional Abundance of the Intestinal Microbiota

**Figure 12:**
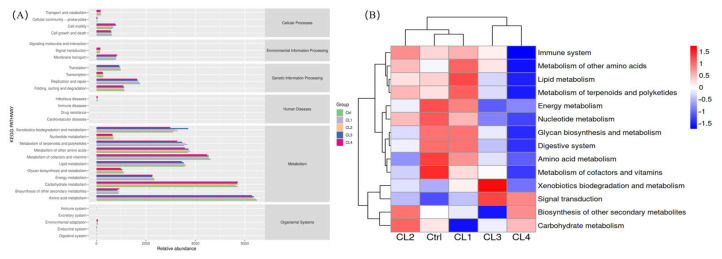
(**A**) Functional prediction of the intestinal microbiome in *L. vannamei* using KEGG pathways. (**B**) Anticipation of the primary metabolic functions of the intestinal microbiome in *L. vannamei* under varying *C. lentillifera* supplementation levels, through KEGG level 2 pathway analysis.

The functional abundance enrichment mapping of the intestinal microbiota (Figure 12) shows enrichment of major metabolic pathways with major emphasis on amino acid metabolism, carbohydrate metabolism, cofactor and vitamin metabolism, lipid metabolism, other amino acid metabolism, metabolism of terpenoid and polyketide, xenobiotics biodegradation and metabolism and energy metabolism.

The cluster thermogram analysis of primary metabolic pathways revealed an increased proportion of pathways associated with lipid metabolism, glycan biosynthesis, and metabolism increased in the CL2 and CL4 groups. Similarly, an elevated proportion of these pathways was observed in CL1, CL2 and CL3 groups. Furthermore, pathways related to metabolism of other amino acids, as well as xenobiotics biodegradation and metabolism, exhibited an increased across all groups supplemented with *C. lentillifera*. Additionally, pathways associated with signal transmission and immune system showed an increased proportion in the CL1 and CL2 groups.

### 3.7. The Relationship Between Intestinal Microbiota and Antioxidant Gene Expression

**Figure 13:**
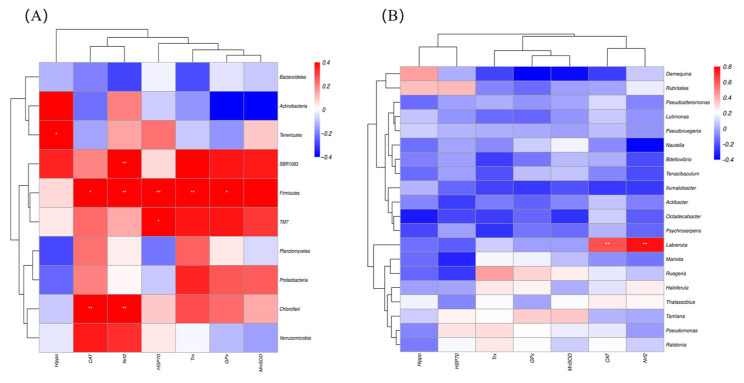
The relationship between intestinal microbiota and antioxidant gene expressions in both the hepatopancreatic and intestines of *L. vannamei* following *C. lentillifera* supplementation. (**A**) Correlation between TOP 10 intestinal microbiota phyla and antioxidant gene expressions in both hepatopancreatic and intestine. (**B**) Correlation between TOP 20 intestinal microbiota genera and antioxidant gene expressions in both hepatopancreatic and intestine. Different asterisk symbols represent statistically significant differences: * corresponds to *p* < 0.05, and ** to *p* < 0.01.

The correlation analysis results between hepatopancreatic antioxidant genes and intestinal microbiota at the Top 10 phylum level (Figure 13A) showed that: Firmicutes exhibited an extremely significant positive correlation with the expression levels of *Nrf2*, *HSP70*, and Trx (*p* < 0.01), and a significant positive correlation with the expression levels of *CAT* and *GPx* (*p* < 0.05). Chloroflexi had an extremely significant positive correlation with the expression levels of *CAT* and *Nrf2* (*p* < 0.01). SBR1093 showed an extremely significant positive correlation with *Nrf2* expression (*p* < 0.01). Tenericutes displayed a significant positive correlation with *Hippo* expression (*p* < 0.05). TM7 exhibited a significant positive correlation with *HSP70* expression (*p* < 0.05). At the Top 20 genus level (Figure 13B), *Labrenzia* had an extremely significant positive correlation with the expression levels of *CAT* and *Nrf2* (*p* < 0.01).

## 4. Discussion

In the context of aquaculture, FCR is a critical metric that indicates the amount of feed required to produce a unit weight of shrimp, serving as a fundamental measure of feed utilization efficiency [26]. PER is an essential indicator, reflecting the efficiency of protein utilization in feeds and thereby informing the selection and optimization of feeds in aquaculture [27]. Previous research has demonstrated that the incorporation of nutritional supplements, such as *spirulina*, can markedly enhance the PER in fish and shrimp, thereby improving protein utilization efficiency [27]. Additionally, the inclusion of *Ulva* meal has been shown to enhance the FCR and PER in Nile tilapia (*Oreochromis niloticus*) [28,29]. Nonetheless, marine crustaceans exhibit limited adaptability to seaweed consumption. Certain studies have indicated that substituting 20% of fishmeal with *Chaetomorpha* alga in the diets of *Penaeus monodon* can improve SR and growth performance, whereas a substitution rate of 30% significantly reduces SR [30]. Similarly, the addition of *C. lentillifera* at levels up to 7.5% did not significant affect the SR of *L. vannamei* in the current study. Our findings indicated that *C. lentillifera* algal meal can enhance feed and protein utilization efficiency in *L. vannamei* when included in appropriate quantities. While growth performance indicators across all groups remained unaffected, inclusion levels up to 10% adversely impacted SR, FCR and PER. The contribution of *C. lentillifera* to FCR and PER exhibited an initial increase followed by stabilization or a potential decrease, suggesting the need for further investigation into optimal dietary inclusions for shrimp. In this study, quadratic regression analyses of FCR and PER identified the optimum inclusion levels of *C. lentillifera* in *L. vannamei* diets as 7.15% and 5.25%, repectively.

The incorporation of *C. lentillifera* up to 7.5% was correlated with an elevation in the crude protein content of *L. vannamei*. These findings align with previous research demonstrating enhanced crude protein content in *L. vannamei* with *C. lentillifera* polysaccharide supplementation and in the muscle composition with brown macroalgae extract (1.5%) and *Spirulina* (5–10%). However, excessive *Spirulina* supplementation (>15%) may impede digestion due to its high fiber content [20,31,32]. Shrimp growth is predominantly facilitated by protein synthesis, which is regulated by the mammalian target of rapamycin (mTOR) signaling pathway. This pathway is responsive to nutrient availability, energy sufficiency, stress, hormones and mitogens, thereby modulating protein synthesis [33,34]. The primary downstream targets of mTORC1’s are components of the translational machinery, notably 4EBP and the 40S ribosomal protein S6K, both of which play crucial roles in the physiological regulation of translation initiation [35,36]. *S6K* is a direct target of mTORC1 and facilitates cell growth by modulating ribosome biosynthesis and translation efficiency [37]. In shrimp, the phosphorylation level of S6K is positively correlated with mTOR activity, and its activation promotes the phosphorylation of ribosomal protein S6, thereby enhancing mRNA translation. The findings of the present study align with these observations. Under conditions of sufficient nutrition, mTORC1 phosphorylates 4EBP, leading to the release of eIF4E-1a, while S6K enhances the activity of its deconjugating enzyme by activating eIF4B, collectively promoting translation efficiency [38]. Correlation analyses revealed that the crude protein content of the whole shrimp exhibited a positive correlation with s6k and eIF4e-1a, indicating that both pathways are the basis for protein synthesis in the shrimp, yet there was no significant difference in the genes related to protein synthesis among the all groups, suggesting that the dietary *C. lentillifera* powder exerted a limited effect on protein synthesis genes of *L. vannamei*.

Antioxidants, including enzymatic (SOD, CAT and glutathione reductase) and non-enzymatic (polyphenols, glutathione and carotenoids) possess the potential to counteract reactive oxygen species (ROS), in turn alleviating oxidative stress and associated health risks. The antioxidant capacity of macroalgae is ascribed to the existence of compounds such as carotenoids, specific polysaccharides, and scavenging-active polyphenols, which exhibit a high affinity for oxidative compounds and can neutralize these reactive oxygen species [39]. Numerous studies have documented that *C. lentillifera* enhances the antioxidant capacity of aquatic organisms [8,40,41]. Our study demonstrated that a diet supplemented with *C. lentillifera* enhanced the activity of antioxidant-related enzymes in *L. vannamei*. SOD, one of the primary antioxidant enzymes, converts superoxide anion (O^2-^) into hydrogen peroxide (H_2_O_2_), while POD facilitates the decomposition of H_2_O_2_ into water, serving as the secondary line of antioxidant defense following SOD [42,43]. MnSOD, a metalloenzyme located in the mitochondria, protects cells from oxidative stress by scavenging ROS [44,45]. CAT and GPx catalyze the conversion of H_2_O_2_ into water [46,47]. MDA is the final product of lipid peroxidation, primarily generated through the oxidative cleavage of polyunsaturated fatty acids (PUFAs) under free radicals attack [48]. MDA serves as a crucial indicator of oxidative stress levels [49]. The high-density conditions and deteriorating water quality associated with intensive aquaculture increase shrimp susceptibility to oxidative stress. Excessive ROS production can overwhelm the antioxidant system, resulting in heightened lipid peroxidation and elevated MDA levels [48]. In prior research, dietary supplementation with 0.1% *C. lentillifera* polysaccharides in shrimp haemolymph resulted in elevated SOD levels and reduced MDA levels. Similarly, the inclusion of *Sargassum aquifolium* in the diet of *O. niloticus* enhanced CAT, SOD and GSH activities while decreasing hepatic MDA levels [50]. Additionally, a low dose of *Enteromorpha* polysaccharides significantly increased T-AOC, SOD, and GPx activities in the haemolymph of *Fenneropenaeus merguiensis* and reduced MDA content [51]. Furthermore, diets enriched with the carotenoid pigment canthaxanthin were found to increase T-AOC and POD activities in *L. vannamei* [52]. Our findings align with these studies, as hepatopancreas antioxidant-related enzyme activities were significantly elevated in both the CL2 and CL3 groups compared to the Ctrl group, whereas MDA levels were significantly reduced. A negative correlation was observed between MDA levels and antioxidant enzyme activities, indicating that 5% *C. lentillifera* markedly enhanced antioxidant capacity and mitigated lipid peroxidation in *L. vannamei*.

Nrf2 serves as a pivotal transcription factor for the antioxidant response element (ARE), facilitating the expression of a diverse array of antioxidant and detoxification genes via the Keap1-Nrf2-ARE pathway. Upon cellular exposure to oxidative or electrophilic stimuli, Nrf2 is dissociates from the Keap1 complex and binds to the ARE, thereby initiating the transcription of downstream genes such as *MnSOD*, *GPx*, and *CAT* [53,54,55,56]. In zebrafish, for instance, Nrf2 modulates the expression of genes encoding antioxidant enzyme in response to oxidative stress and environmental toxicant-induced damage through the Keap1-Nrf2 signaling pathway [53,57]. The silencing of Nrf2 has been shown to markedly diminish the expression of antioxidant-related genes, including *SOD*, *GPx*, *CAT*, *Trx*, and *HO-1*, indicating Nrf2’s involvement in the antioxidant capacity and oxidative stress response of *L. vannamei* [58,59]. MnSOD, GPx, and CAT collaboratively function to scavenge distinct ROS, while Trx repairs oxidative damage, collectively maintaining cellular redox homeostasis [60,61]. Additionally, HSP70, a heat shock protein, alleviates oxidative stress-induced cellular damage by facilitating the repair and degradation of misfolded proteins [62]. The Hippo signaling pathway potentially influences the expression of antioxidant enzymes by modulating cellar proliferation and apoptosis [63]. Within the context of immune responses, antimicrobial peptides regulated by the Hippo pathway work synergistically with enzymes such as GPx to combat pathogenic infections and mitigate infection-induced oxidative stress [64,65]. The results indicated that there was no significant difference in the expression levels of antioxidant genes, suggesting that the dietary *C. lentillifera* powder exerted a limited effect on the hepatopancreas of *L. vannamei* at the molecular level. While significant differences were observed in the activities of antioxidant enzymes, which might be attributed to multi-level regulations such as translation efficiency, post-translational modifications, and cofactor availability [66,67,68]. These regulatory factors can directly enhance or inhibit the activities of antioxidant enzymes, even when no significant changes occur in the expression levels of their corresponding antioxidant genes. Notably, *Nrf2* expression was positively correlated with *CAT* expression in the correlation analysis results, implying a pivotal role for *Nrf2* in regulating *CAT* expression following the introduction of algal powder. The relationship between antioxidant-related enzyme activities and gene expressions indicated that *Nrf2*, *CAT*, *Trx* and *HSP70* expressions remain the basis for the enhancing effect of T-AOC on the compensatory capacity against oxidative damage, and *HSP70* and *Hippo* expressions remain the basis for the reduction in MDA conten.

The animal intestine is a critical organ, with most of its function, such as immunity, health regulation, and nutrient absorption, being mediated through bacterial metabolism [69,70]. Optimizing dietary formulations or supplementing with additives presents a viable strategy for modulating the intestinal microbiota in shrimp [69]. In the investigation of intestinal microbiota, 16S rRNA sequencing technology serves as a fundamental analytical tool, enabling precise analysis of the composition and diversity of intestinal microbiota. The technique facilitates accurate taxonomic identification of microbial communities through sequencing, and allows for the quantification of abundance and structural variations within the microbiota using α-diversity indices (such as Chao1, Shannon and Simpson indices) and β-diversity measures (such as PCoA analysis). Furthermore, it elucidates the impact of diseases or interventions on the intestinal microbiota. The α-diversity analysis in this study revealed that the inclusion of *C. lentillifera* at levels up to 7.5% enhanced the diversity (Shannon and Simpson) and evenness (Pielou_e) of the intestinal microbiota. Notably, the highest diversity was observed at a 7.5% inclusion level, suggesting that the intestinal ecosystem may achieve greater stability at this concentration. A decline in these metrics was detected in the CL4 group, indicating that high doses (10%) of *C. lentillifera* may exert adverse effects.

The Bray–Curtis-based β-diversity analysis revealed structural differences in the intestinal microbiota communities across the groups, suggesting that the inclusion of *C. lentillifera* altered the distribution and composition of the intestinal microbiota. Analysis of the community composition at the phylum level identified Proteobacteria, Bacteroidetes, Actinobacteria, and Verrucomicrobia as the predominant bacterial taxa within the intestinal microbiota of *L. vannamei*. A recent study has demonstrated that the phyla Proteobacteria, Bacteroidetes, and Actinobacteria predominantly constitute the intestinal microbiota community of *L. vannamei* across all developmental stages. Among these, Proteobacteria emerged as the most central and stable component, maintaining consistent abundance regardless of environmental or dietary change [69]. The findings of the current study further corroborate the central role of these bacteria across three phyla. Proteobacteria, in particular, dominate the shrimp intestine and exhibit significant diversity and multifunctionality, contributing to essential physiological processes such as the nitrogen cycle, carbon-sulfur cycle, enzyme secretion, metabolism, and immunomodulation [71]. As the principal Gram-negative bacterial group in the shrimp intestine, Proteobacteria preferentially utilize protein as their primary energy source and produce proteases [72]. The relative abundance of Proteobacteria and Firmicutes, in the intestinal microbiota of *L. vannamei* when *Chlorella sorokiniana* was employed as the main protein source, aligning with the results of our study [73]. Firmicutes play a crucial role in the intestinal microbiota of shrimp, predominantly influencing energy metabolism. They possess numerous genes responsible for the fermentation of dietary fiber [74], excelling in the degradation of complex polysaccharides, such as cellulose, and the generation of short-chain fatty acids (SCFAs). This process not only facilitates the absorption and storage of energy by the host but also enhances intestinal barrier function and inhibits the proliferation of pathogenic bacteria [75,76]. Greater abundance of Firmicutes correlates with an increase in lipid droplet count, thus proportionally boosting host uptake of fatty acids [77]. The Firmicutes/Bacteroidetes (F/B) ratio is considered to be significantly correlated with gut microbiota composition [76], where a high F/B ratio indicates improved intestinal transport and digestive capacity [78], as well as in increased ability to extract energy from feed [79]. In this study, the F/B ratio initially increased and then decreased following the addition of algal meal, yet remained higher than the control throughout. In conjunction with observed alterations in growth performance, these findings imply that algal meal may enhance nutrient digestion and absorption resulting in a reduced FCR in shrimp, aligning with previous results [80].

*Demequina* exhibits probiotic effects in the intestine primarily through the degradation of complex organic matter, involvement in elemental cycling, and pathogen inhibition. However, it has been demonstrated that a significant increase in the relative abundance of *Demequina* in *L. vannamei* is positively correlated with H_2_O_2_ and P53, while negatively correlated with metabolites under microcystin-LR stress [81], indicating that *Demequina* may disrupt host metabolic homeostasis. The marked reduction in Demequina in the CL3 group indicates that the incorporation of 7.5% *C. lentillifera* may alleviate the potential risks associated with *Demequina*. *Rubritalea*, a number of the Verrucomicrobia phylum, is prevalent in the marine environments and the intestines of aquatic animals [82,83]. It produces carotenoids and squalene, which are potentially linked to antioxidant or photo-protective functions [83,84]. The carotenoids synthesized by *Rubritalea* may neutralize ROS in the intestinal tract, thereby reducing oxidative stress and safeguarding host cells. Squalene, a precursor in cholesterol synthesis, may contribute to cell membrane stabilization and immune signaling in shrimp [84]. In experiments utilizing rice protein as substitute protein source, the abundance of *Rubritalea* in shrimp initially increased and subsequently decreased, mirroring the findings of the current study [80].

Intestinal microbiota is crucial in regulating physiological processes in the host and exert a vital role in promoting as well as maintaining host health [85]. *Pseudoruegeria*, a member of the phyla Proteobacteria, and class Alphaproteobacteria, has not been studied in the context of the shrimp intestine. However, it shares a close phylogenetic relationship with the genera *Ruegeria* and *Silicibacter* [86]. *Ruegeria* species are known to engage in symbiotic relationships with their hosts, contributing to carbohydrate degradation and VB_12_ synthesis, processes that are positively correlated with shrimp growth rate [87,88]. Genomic analyses of certain *Pseudoruegeria* strains, such as M32A2M, have demonstrated the presence of complete pathways for the synthesis of VB_1_, VB_7_, and VB_12_ [89]. These pathways are associated with the upregulation of glycolysis and tricarboxylic acid cycle-related genes, enhanced utilization of host-derived undigested carbon sources, secretion of peptide transporter proteins, and promotion of amino acid uptake [90]. The notable increase in the abundance of *Pseudoruegeria* in the intestinal tract of *L. vannamei* following exogenous glucose supplementation is likely attributable to its metabolic capacity, which is well-suited to a carbon rich environment [88]. Recent research indicates that *Pseudoruegeria* could act as a biomarker associated with the degradation of algal cell wall polysaccharides [91]. In this study, emphasis was placed on the phyla Proteobacteria and Bacteroidetes, as well as the genera *Demequina* and *Rubritalea*. The results suggest that *C. lentillifera* may play a role in increasing the relative abundance of these beneficial intestinal microbiota, which potentially promote nutrient absorption and metabolism, boost immune function, inhibit pathogenic bacteria.

The results of the correlation analysis suggest a certain association between the expression of hepatopancreatic antioxidant genes and the intestinal microbiota. Previous studies have also confirmed that Firmicutes has a clear link to antioxidant function, and changes in Firmicutes can affect the host’s oxidative stress levels. Firmicutes encompasses a variety of probiotics, which do not directly exhibit “antioxidant activity” themselves. However, they can enhance the overall antioxidant capacity by producing SCFAs and upregulating the expression of antioxidant enzyme genes (*SOD*, *CAT*, *GPx*) in the host intestine [92,93]. Alternatively, Firmicutes may regulate the antioxidant stress phenotype of the gut microbiota, which could potentially upregulate the expression of *SOD* and *CAT* genes in the host, thereby reducing MDA levels [94]. SBR1093, a candidate genus within Firmicutes [95], may be involved in metabolic regulation. In contrast, no antioxidant effects of Tenericutes, Chloroflexi, TM7, and *Labrenzia* have been identified in the existing research to date. This finding indicates that in future studies, we can pay more attention to the associations between these intestinal microbiota and antioxidant genes, and understand the mechanisms of host–microbe interactions from a more systematic and integrated perspective.

For the intestinal microbiota of *L. vannamei*, functional predictions demonstrate notable enrichment in metabolic pathways. Specifically, supplementation with low doses of *C. lentillifera* generally elevated the proportion of secondary pathways related to carbohydrate metabolism, lipid metabolism, metabolism of other amino acids, xenobiotics biodegradation and metabolism, signal transduction, and the immune system. The augmentation in the proportion of metabolic pathways suggests that *C. lentillifera* may enhance nutrient utilization efficiency, improve energy metabolism, bolster the organism’s detoxification capacity against external harmful substances, optimize the intercellular communication mechanisms, and strengthen the immune defense system. Consequently, this enhances the organism’s adaptive capabilities and health of the organism.

## 5. Conclusions

The incorporation of *C. lentillifera* into the diet of *L. vannamei* is scientifically justified. The inclusion of 2.5–7.5% *C. lentillifera* has been demonstrated to enhance growth performance, antioxidant capacity, and the structure of intestinal microbiota in *L. vannamei*. On the basis of the findings from this study, it is advisable to include *C. lentillifera* at a concentration of 5.25–7.15% in the diet of *L. vannamei*.

## Figures and Tables

**Table 1:** Ingredients and proximate composition of the different experimental diets (%).

Ingredients ^a^	Ctrl	CL1	CL2	CL3	CL4
*C. lentillifera* ^b^	0.00	2.50	5.00	7.50	10.00
Fish meal	25.00	25.00	25.00	25.00	25.00
Soybean meal	18.00	18.00	18.00	18.00	18.00
Peanut meal	16.40	16.40	16.40	16.40	16.40
Wheat flour	24.00	14.00	14.00	14.00	14.00
Bentonite	0.00	7.50	5.00	2.50	0.00
Beer yeast	5.00	5.00	5.00	5.00	5.00
Krill meal	5.00	5.00	5.00	5.00	5.00
Soy lecithin	1.00	1.00	1.00	1.00	1.00
Fish oil	1.00	1.00	1.00	1.00	1.00
Soybean oil	1.00	1.00	1.00	1.00	1.00
Choline chloride (50%)	0.50	0.50	0.50	0.50	0.50
Dicalcium phosphate	1.00	1.00	1.00	1.00	1.00
Multi-minerals ^c^	1.00	1.00	1.00	1.00	1.00
Multi-vitamins ^d^	1.00	1.00	1.00	1.00	1.00
Vc phosphate	0.10	0.10	0.10	0.10	0.10
Sum	100.00	100.00	100.00	100.00	100.00
Nutrient levels (dry weight)					
Moisture	7.34	8.25	8.51	7.45	7.90
Ash	13.36	15.72	15.70	14.43	14.07
Crude protein	45.27	44.47	44.69	45.77	46.36
Crude lipid	10.96	10.92	11.08	11.59	11.61

Note: ^a^ Fish meal, soybean meal, wheat flour, krill meal, peanut meal, beer yeast, fish oil, dicalcium phosphate, choline chloride, and Vc phosphate, purchased from Qingdao Baiwei Yingge Biotechnology Co., Ltd. (Qingdao, China). ^b^ Purchased from Shenzhen Lantene Biological Technology Co., Ltd. (Shenzhen, China). ^c^ Purchased from Guangzhou Xinhailisheng Biotechnology Co., Ltd. (Guangzhou, China). Mineral premix (g kg^−1^): KCl, 90; NaCl, 40; KI, 0.04; ZnSO_4_·7H_2_O, 4; CuSO_4_·5H_2_O, 3; CoSO_4_·7H_2_O, 0.02; MnSO_4_·H_2_O, 3; FeSO_4_·7H_2_O, 20; MgSO_4_·7H_2_O, 124; CaCO_3_, 215; Ca(H_2_PO_4_)_2_·2H_2_O, 500. ^d^ Purchased from Guangzhou Bauxite Aquatic Technology Co., Ltd. (Guangzhou, China). Vitamin premix (g kg^−1^): VE, 75; VK, 2.5; VB_1_, 0.25; VB_2_, 1.0; VB_3_, 5.0; VB_6_, 0.75; VB_12_, 2.5; VA, 2.5; VD, 6.25; VB_9_, 0.25; cellulose, 500; VB_h_, 379; VB_7_, 2.5.

**Table 2:** Effects of diets adding *C. lentillifera* on the growth performance of *L. vannamei.*

Items	Ctrl	CL1	CL2	CL3	CL4
IBW ^1^ (g)	2.53 ± 0.03	2.42 ± 0.08	2.50 ± 0.06	2.42 ± 0.06	2.51 ± 0.05
FBW ^2^ (g)	11.07 ± 0.27	9.89 ± 0.17	10.60 ± 0.51	10.80 ± 1.07	10.57 ± 0.43
SR (%)	99.17 ± 1.66	100.00 ± 0.00	95.83 ± 3.19	94.17 ± 5.00	92.50 ± 5.00
WGR (g)	337.31 ± 7.80	309.72 ± 19.62	324.57 ± 22.64	344.92 ± 34.25	321.62 ± 18.93
SGR (% d^−1^)	2.73 ± 0.03	2.61 ± 0.09	2.67 ± 0.10	2.76 ± 0.14	2.66 ± 0.08
FCR	1.19 ± 0.04	1.19 ± 0.03	1.11 ± 0.07	1.11 ± 0.06	1.15 ± 0.04
PER	1.86 ± 0.06	1.90 ± 0.04	2.04 ± 0.13	1.96 ± 0.11	1.87 ± 0.06

Note: ^1^ Initial body weight (IBW, g); ^2^ Final body weight (FBW, g).

**Table 3:** Effects of dietary supplementation with *C. lentillifera* on the body composition of *L. vannamei* (dry weight, %).

Items	Ctrl	CL1	CL2	CL3	CL4
Moisture	76.63 ± 0.49	76.11 ± 0.19	76.37 ± 0.83	76.03 ± 0.43	76.11 ± 0.50
Crude protein	75.70 ± 0.26 ^c^	78.55 ± 0.16 ^a^	78.32 ± 0.15 ^ab^	77.99 ± 0.21 ^b^	77.20 ± 1.03 ^abc^
Crude lipid	4.77 ± 0.41	4.77 ± 0.56	4.83 ± 0.74	4.37 ± 0.69	3.97 ± 0.67
Ash	12.52 ± 0.50	12.42 ± 0.64	12.57 ± 0.81	12.83 ± 0.57	13.36 ± 0.74

Note: Means with different superscripts are significantly different (*n* = 4, *p* < 0.05).

**Table 4:** Effects of dietary supplementation with *C. lentillifera* on hepatopancreas antioxidant enzyme activities of *L. vannamei*. (U mg prot^−1^).

Items	Ctrl	CL1	CL2	CL3	CL4
T-AOC	0.10 ± 0.02	0.14 ± 0.02	0.27 ± 0.01	0.28 ± 0.01	0.09 ± 0.01
T-SOD	7.58 ± 1.24 ^c^	14.60 ± 1.15 ^ab^	12.32 ± 0.97 ^b^	15.50 ± 0.60 ^a^	13.88 ± 1.68 ^ab^
CAT	0.10 ± 0.02	0.11 ± 0.03	0.14 ± 0.02	0.15 ± 0.01	0.15 ± 0.01
GPx	185.82 ± 12.02 ^d^	353.47 ± 9.24 ^a^	328.83 ± 3.52 ^b^	234.04 ± 5.92 ^c^	132.38 ± 3.86 ^e^
MDA	1.79 ± 0.42 ^a^	1.05 ± 0.10 ^a^	0.43 ± 0.03 ^b^	1.13 ± 0.31 ^ab^	1.18 ± 0.21 ^a^
POD	1.52 ± 0.20 ^b^	1.45 ± 0.32 ^b^	1.86 ± 0.13 ^ab^	2.12 ± 0.25 ^a^	2.26 ± 0.27 ^a^

Note: Means with different superscripts are significantly different (*n* = 4, *p* < 0.05).

## Data Availability

Data will be made available upon request.

## Supplementary Materials

The following supporting information can be downloaded at: https://www.mdpi.com/article/10.3390/biology14121738/s1, Table S1. Nutritional components of *C. lentillifera* cultivated in Dapeng Bay (*n* = 3, dry weight, %).; Table S2: Primers for Real-time fluorescence quantitative PCR.; Table S3: Composition of the top 10 microbial phyla in the intestine contents of *L. vannamei* across different treatment groups (*n* = 4); Table S4: Composition of the top 10 microbial genera across the intestine contents of *L. vannamei* across different treatment groups (*n* = 4).
