# Supplementary material for: Effects of Caulerpa lentillifera on Growth Performance, Antioxidant Capacity and Intestinal Microbiota of Litopenaeus vannamei
Source: Biology (Basel). 2025 Dec 4;14(12):1738. doi: 10.3390/biology14121738 (PMC12730858; doi:10.3390/biology14121738)
Supplement: Supplementary file 1 [file biology-14-01738-s001.zip › biology-3987383-supplementary.pdf]

Table S1. Nutritional components of *C. lentillifera* cultivated in Dapeng Bay (n = 3, dry weight, %)

Items	*C. lentillifera*
Moisture	94.47 ± 0.51
Ash	34.07 ± 1.34
Crude protein	18.70 ± 0.36
Crude lipid	4.57 ± 0.47

Note: the nutritional components of *C. lentillifera* were measurement by Zhou [17].

Table S2. Primers for Real-time fluorescence quantitative PCR.

Genes	Forward Primer	Reverse Primer	GenBank Nos.
Nrf2	TCTTGTTGGTCCCTCGCTCCTC	TCACTGCTTGGGGTCATCCTTCC	XM_027367070.1
GPx	GGCACCAGGAGAACACTAC	CGACTTTGCCGAACATAAC	AY973252.2
CAT	TACTGCAAGTTCCATTACAAGACG	GTAATTCTTTGGATTGCGGTCA	XM_027383088.1
MnSOD	AAAGCATCACCAGGGCTACAT	CGCCTCCATTGAACTTGATAG	XM_070117072.1
Trx	TCATCAATCAGCCGCATACCATCG	CATCGTCCGACTGTCCACTTCATC	XM_070125473.1
Hippo	TGAGCACAACCAAACCCACCATC	CATCGTCCGACTGTCCACTTCATC	MW415984.1
HSP70	AGGAGACCGCTGAGGCTTAC	AGCACATTCAGACCCGAGAT	XM_027369405.2
mtor	CGAACACTCGGGTCCTTTGA	GAGGTGACAGCAAGTTCGGA	XM_027372359.2
s6k	GCAAGAGGAAGACGCCATA	CCGCCCTTGCCCAAAACCT	XM_027368997.2
4ebp	ATGTCTGCTTCGCCCGTCGCTCGCC	GGTTCTTGGGTGGGCTCTT	XM_027367939.2
eif4e-1a	TGGAATCAAACCTATGTGGG	GTCCTCCTGGAAGCGTA	XM_027354395.2
β-actin	GCCCTGTTCCAGCCCTCATT	ACGGATGTCCACGTCGCACT	XM_027364954.2

Table S3. Composition of the top 10 microbial phyla in the intestine contents of *L. vannamei* across different treatment groups (n = 4).

Items	Ctrl	CL1	CL2	CL3	CL4
Proteobacteria	54.47 ± 6.74 ^{b}	57.82 ± 7.01 ^{b}	56.8 ± 5.38 ^{b}	70.08 ± 9.41 ^{a}	70.94 ± 6.38 ^{a}
Bacteroidetes	31.28 ± 8.87 ^{ab}	32.81 ± 8.88^{a}	20.65 ± 6.99 ^{abc}	17.58 ± 4.8 ^{bc}	15.75 ± 12.42 ^{c}
Actinobacteria	13.1 ± 5.43 ^{a}	7.92 ± 2.76 ^{ab}	12.69 ± 3.9 ^{a}	6.03 ± 2.95 ^{b}	8.32 ± 3.48 ^{ab}
Verrucomicrobia	0.77 ± 0.65	1.04 ± 1.19	4.44 ± 3.35	3.41 ± 2.87	2.45 ± 3.97
Tenericutes	0.06 ± 0.05	0.12 ± 0.19	4.84 ± 5.75	0.49 ± 0.53	2.04 ± 3.23
Firmicutes	0.05 ± 0.03 ^{b}	0.06 ± 0.01 ^{b}	0.08 ± 0.02 ^{b}	0.77 ± 0.63 ^{a}	0.04 ± 0.03 ^{b}
Planctomycetes	0.12 ± 0.1 ^{ab}	0.04 ± 0.03 ^{b}	0.15 ± 0.17 ^{ab}	0.39 ± 0.33 ^{a}	0.26 ± 0.25 ^{ab}
Chloroflexi	0.01 ± 0.01 ^{b}	0.04 ± 0.01 ^{b}	0.03 ± 0.01b	0.47 ± 0.52 ^{a}	0.01 ± 0.01 ^{b}
TM7	0.01 ± 0.02	0.01 ± 0.01	0.09 ± 0.17	0.15 ± 0.24	0.08 ± 0.14
SBR1093	0.02 ± 0.03	0.04 ± 0.03	0.06 ± 0.09	0.12 ± 0.12	0.01 ± 0.03

Table S4. Composition of the top 10 microbial genera in the intestine contents of *L. vannamei* across different treatment groups (n = 4).

Items	Ctrl	CL1	CL2	CL3	CL4
Nautella	28.84 ± 5.85	30.88 ± 6.17	29.33 ± 9.81	24.78 ± 5.68	26.72 ± 20.48
Ruegeria	13.11 ± 7.48	10.3 ± 2.82	12.86 ± 3.73	19.34 ± 6.75	13.78 ± 9.95
Demequina	8.85 ± 5.28 [a]	6.94 ± 2.85 [ab]	9.13 ± 5.44 [a]	2.02 ± 2.06 [b]	4.31 ± 2.33 [ab]
Paracoccus	0.00 ± 0.00	0.00 ± 0.00	0.05 ± 0.09	0.00 ± 0.00	16.45 ± 32.9
Octadecabacter	3.29 ± 2.29 [ab]	5.41 ± 2.86 [a]	1.91 ± 1.55 [b]	2.29 ± 1.62 [ab]	1.37 ± 1.34 [b]
Shimia	0.54 ± 0.46	1.19 ± 0.79	1.12 ± 1.00	3.58 ± 4.49	1.88 ± 1.24
Thalassobius	0.54 ± 0.26	0.44 ± 0.22	1.26 ± 0.74	1.35 ± 0.51	1.45 ± 1.32
Ralstonia	0.02 ± 0.02	0.05 ± 0.07	0.24 ± 0.33	1.36 ± 1.37	2.19 ± 2.84
Psychroserpens	0.84 ± 0.56 [ab]	1.5 ± 0.94 [a]	0.64 ± 0.58 [ab]	0.47 ± 0.29 [b]	0.17 ± 0.19 [b]
Rubritalea	0.11 ± 0.15 [b]	0.75 ± 1.1 [ab]	1.59 ± 0.93 [a]	0.68 ± 0.37 [ab]	0.15 ± 0.17 [b]
